# McArdle’s Disease: A Differential Diagnosis of Metabolic Myopathies

**DOI:** 10.7759/cureus.70000

**Published:** 2024-09-23

**Authors:** Joana Nascimento, Raquel Pinho, Ana Pimenta de Castro, Nuno Bernardino Vieira

**Affiliations:** 1 Internal Medicine, Centro Hospitalar Universitário do Algarve, Unidade de Portimão, Portimão, PRT; 2 Internal Medicine, Unidade Local de Saúde do Algarve, Unidade Hospitalar de Portimão, Portimão, PRT; 3 Internal Medicine, Unidade Local de Saúde do Algarve, Unidade Hospitalar de Faro, Faro, PRT

**Keywords:** rhabdomyolysis, myoglobinuria, exercise intolerance, glycogen storage disease type v, mcardle's disease

## Abstract

McArdle's disease, also known as glycogen storage disease type V or McArdle syndrome, is a pure muscle myopathy with an autosomal recessive inheritance pattern. It is caused by mutations in the gene that encodes muscle phosphorylase. Symptoms typically begin in late adolescence or early adulthood, presenting as exercise intolerance. This review focuses on the diagnosis of McArdle's disease, initially manifesting as a clinical picture of rhabdomyolysis in an 18-year-old male patient with a history of minor thalassemia who had been followed in pediatric consultation since age three for failure to thrive.

After excluding common causes such as alcohol consumption, drug use, traumatic muscle compression, and other conditions, the diagnosis of McArdle's disease was considered. The diagnosis was supported by laboratory tests showing myoglobinuria and elevated creatine kinase levels, as well as the absence of increased serum lactate following ischemic exercise. Genetic testing confirmed the presence of mutations in the PYGM gene, corroborating the diagnosis.

Treatment includes administering a diet rich in slow-absorbing carbohydrates, regular low-intensity physical exercise, and, in some cases, supplementation with vitamin B6 and creatine. The prognosis is generally favorable with proper disease management, although vigorous exercise should be avoided to prevent complications such as severe muscle injury and rhabdomyolysis.

Although McArdle's disease is a rare condition, it is likely underdiagnosed. Ideally, it should be considered in the differential diagnosis of rhabdomyolysis in all patients with symptoms of exercise intolerance and/or recurrent myoglobinuria.

## Introduction and background

McArdle's disease, also known as glycogen storage disease type V, or muscle phosphorylase deficiency, is a rare metabolic disorder first described in 1951 by Brian McArdle, with the underlying enzymatic alteration identified in 1959 by Schmid and Mahler [1-7]. The exact incidence of this glycogen storage disease is unknown due to its benign nature and late or even underdiagnosis [8], with an estimated prevalence of 1:100,000-1:167,000 [9-11].

It is characterized by an accumulation of glycogen in skeletal muscles, caused by a deficiency of myophosphorylase, the muscle isoform of the glycogen phosphorylase enzyme, which limits the production of adenosine triphosphate (ATP) through glycogenolysis [3,6-7].

This glycogen storage disease is a genetic disorder, predominantly inherited in an autosomal recessive manner, although rare cases of symptomatic heterozygosity are described [6,12-13]. The disease is caused by mutations in both alleles of the PYGM gene (muscle glycogen phosphorylase), which encodes myophosphorylase, located on chromosome 11q13 [3-6,11].

The R50X mutation in exon 1 is the most common mutation in the Caucasian population, with higher expression in Northern Europe and North America, and there is global evidence of more than 100 distinct mutations, with no identified genotype-phenotype correlation to date [6,8,11,14], and the human genetic mutation database lists 131 distinct mutations [15].

The R50X mutation results in the replacement of a cytosine with a thymine at codon 50 in exon 1, leading to the introduction of a STOP codon, which interrupts the normal insertion of the codon for the amino acid arginine (R) [4,6].

Glycogen (muscular and hepatic) is a polysaccharide used for cellular energy storage, composed of glucose subunits, with muscle glycogen being metabolized locally to provide energy for muscle contraction and is therefore converted to lactate [16-17].

Myophosphorylase plays a crucial role in glycogen metabolism, as this enzyme is essential for carbohydrate metabolism and is responsible for degrading glycogen to lactic acid by removing 1,4-α-glucosyl units from the glycogen molecule, releasing glucose-1-phosphate, and subsequently forming ATP [3-5,14,18].

Glycogen synthesis initially requires the action of a hexokinase-glucokinase to convert glucose to glucose-6-phosphate, which is then converted to glucose-1-phosphate by phosphoglucomutase. In turn, glucose-1-phosphate is transformed into uridine diphosphoglucose. This process builds a large, highly branched polymer containing glucose molecules linked by α-1,4-glycosidic bonds, the site of action of myophosphorylase [4,19].

In the presence of myophosphorylase deficiency, an enzyme essential for glycolysis, the α-1,4-glycosidic bonds cannot be removed, preventing glycogen degradation, leading to pathological storage of glycogen in muscles and a related energy deficit [4-5,19].

Given that the PYGM gene is solely responsible for the formation of the muscle isoform of glycogen phosphorylase and that the hepatic and cardiac isoforms are not affected, McArdle's disease is understood to be a pure myopathy [3-4].

McArdle's disease typically presents symptomatically between the ages of 10 and 30, with only 4% of cases being diagnosed before age 10 and rarely after age 30 [7-8,18].

As a consequence of the deficient activity of myophosphorylase, individuals with McArdle's disease are unable to obtain energy from their muscle glycogen reserves [3]. Thus, symptoms of exercise intolerance, such as intermittent myalgias, contractures, fatigue, and muscle weakness during exercise, are the primary manifestations of this disease [8-9]. Typically, symptoms are triggered by brief but intense exercise. However, less intense but prolonged exercise can also provoke symptoms [5-8,14,18-19].

The "second-wind" phenomenon is experienced by individuals with McArdle's disease. This phenomenon, occurring approximately 10 minutes after the onset of exertion, denotes a sudden and significant improvement in aerobic exercise tolerance after a brief rest period, following the development of muscle stiffness and myalgias, enabling the person to continue physical activity with a resolution of symptoms triggered at the start of exertion [3,5-6,9,18,20-21]. The "second wind" can be explained by increased blood flow, optimized delivery of free fatty acids with concurrent activation of their metabolism, and increased utilization of glucose [3,9,21]. However, pre-exercise glucose intake can prevent the onset of this phenomenon [3].

In addition to exercise intolerance, this disease also causes, in about 50% of cases, myoglobinuria with burgundy-colored urine secondary to rhabdomyolysis, which can trigger acute kidney injury in about 27% of these patients [6-8,19], with this being the initial manifestation of the disease in approximately 10% of cases [5].

Clinically, several presentation patterns have been described: the neonatal form, characterized by generalized muscle hypotonia, respiratory failure, and premature death; the mild form, with subtle symptoms such as asthenia or lack of endurance; the classic form, with symptomatic onset in adolescence and early adulthood, evidenced by muscle contractures, myalgias, and myoglobinuria after physical exertion; and the late-onset presentation, after age 40, at which point a change in symptom pattern is observed, with improvement in muscle contractures and myoglobinuria and worsening muscle weakness with evidence of proximal muscle atrophy [4,6,14,16].

This review arises in the context of an 18-year-old male patient with a history of minor thalassemia, followed in pediatric consultation since the age of three for failure to thrive with elevated transaminases, who presented to the emergency department with dark urine after intense physical exertion, without other complaints. Urinalysis revealed myoglobinuria, and biochemical studies demonstrated microcytic and hypochromic anemia (Hb 118 g/L, MCV 58.8 fL, MCH 19.1 pg), leukocytosis (11.7 x10^9/L) with neutrophilia (neutrophils 8.2 x10^9/L), elevated CK-MB isoenzyme levels (213.4 µg/L), elevated (CK 31,696 IU/L), myoglobin (>1200 µg/L), and creatinine (1.40 mg/dL). Lipid profile, thyroid function, and autoimmune tests were normal.

Electromyography (EMG) and muscle biopsy (after EMG) were performed, which did not identify any abnormalities.

The study of mitochondrial and metabolic diseases revealed slightly elevated plasma carnitine levels (total carnitine of 76.7 µM, free carnitine of 48.8 µM, and acylcarnitine of 27.9 µM), normal plasma and urine amino acids, and normal urinary organic acids.

Suspecting a metabolic myopathy, a genetic test for McArdle’s disease was requested along with a concomitant forearm ischemia test. The forearm ischemia test did not show an increase in lactate and pyruvate (suggestive of McArdle's disease), and the definitive diagnosis of McArdle’s disease was established after identifying the mutations c.148C>T (p.R50X) in exon 1 and c.613G>A (p.G205S) in exon 5, both in heterozygosity in the PYGM gene.

Family genetic testing revealed the same heterozygous mutations in both parents. Molecular testing of the mother revealed the presence of the c.613G>A (p.G205S) mutation in exon 5 in heterozygosity, and the father’s test detected the presence of the c.148C>T (p.R50X) mutation in exon 1 in heterozygosity.

Currently, after five years of follow-up, under a hydration plan and increased pre-exercise sucrose intake, the patient remains asymptomatic, denying symptoms of exercise intolerance, and has not experienced new episodes of rhabdomyolysis or myoglobinuria.

## Review

High-intensity physical exercise is prone to triggering reversible symptoms of exercise intolerance, primarily manifested as myalgias, muscle contractions, and early muscle weakness, which can even culminate in a clinical picture of rhabdomyolysis [22-24].

In this patient, the diagnosis of rhabdomyolysis was established by identifying myoglobinuria and elevated plasma muscle enzymes, particularly creatine kinase (CK). Thus, the absence of muscle symptoms alone does not exclude the possibility of this syndrome. Specifically, the presence of myoglobinuria, combined with elevated CK levels five times the normal upper limit, supports the diagnosis of rhabdomyolysis [23-26].

When faced with a case of rhabdomyolysis, it is important to clarify its etiology. In this regard, the most frequently associated causes should be considered, such as alcohol and toxic substance use (drugs and medications), traumatic muscle compression, neuroleptic malignant syndrome, and excessive muscle activity [23-24,26-29].

If elevated CK levels persist after the cessation of exercise, less common etiologies of rhabdomyolysis should be considered, such as conditions that promote muscle hypoperfusion (e.g., thrombosis and embolism), endocrine and electrolyte abnormalities, infectious diseases, and inflammatory and metabolic myopathies [8,14,24-27].

Given the wide range of underlying conditions, it is crucial to establish the association between the type and intensity of exercise performed and the symptoms triggered to infer the level of the defect in muscle energy metabolism [6].

Fatty acids and glycogen are the primary sources of muscle energy, with fatty acids playing a crucial role during rest, low-intensity exercise, and prolonged durations, whereas glycogen is essential for high-intensity, especially short-duration activities [30-32].

The definitive diagnosis of McArdle's disease is established through an enzymatic assay of muscle tissue or mutation analysis of the myophosphorylase gene. However, certain laboratory tests, such as CK level measurement and myoglobinuria, are important indicators of this glycogenosis [4,7].

Regarding serum CK levels, it is important to note that in McArdle's disease, and in more than 90% of cases, these levels remain elevated at rest, which allows differentiation from carnitine palmitoyltransferase II deficiency, where CK levels normalize during asymptomatic phases [7,32-34].

Ammonia and lactate measurements are particularly important in suspected metabolic myopathy cases. The absence of elevated serum lactate combined with disproportionate increases in ammonia levels after physical activity strongly indicates muscle glycogenosis and suggests a defect in converting glycogen or glucose into lactate [4,7,17].

Regarding EMG, the characteristic pattern in patients with McArdle's disease is the absence of electrical activity in the contracted muscle, with a possible observation of myotonic discharges during anaerobic exercise [4,6,32-33]. EMG also helps to pinpoint the location for muscle biopsy [6].

The forearm ischemia test, also known as the ischemic forearm exercise test, introduced by McArdle, has long been considered the gold standard for diagnosing metabolic myopathies. In McArdle's disease, this test presumes an absence of elevation in serum lactate [1,3,6,15,35-36].

This test involves placing a peripheral venous catheter (PVC) and obtaining venous blood samples for lactate, pyruvate, and ammonia levels before exercise, preferably from the antecubital vein, followed by the application of a blood pressure cuff inflated above the patient’s diastolic blood pressure, while the forearm is exercised (vigorously opening and closing the hand) for one minute. A new peripheral venous blood sample is then collected. After exercise cessation, additional venous samples are collected at 2, 3, 5, and 10 minutes [35,37-38].

The lack of increase in serum lactate concentration during exercise suggests a defect in the conversion of glycogen to lactate, consistent with the pathophysiology of McArdle's disease as well as other glycogenoses [36].

It is particularly useful for screening patients with suspected metabolic myopathy before proceeding with more invasive and costly investigations, such as muscle biopsy and genetic testing [35]. However, it is a painful complementary diagnostic test (CDT) with low sensitivity and specificity and potential complications such as local muscle injury with myoglobinuria and, less commonly, compartment syndrome. Therefore, it should be discontinued if the patient experiences myalgias or muscle contractions during the test [1,3,6,15,35].

According to scientific evidence, resting ammonia levels are expected to be low, with a drastic increase after exertion, unlike plasma lactate, which decreases in the post-ischemic effort period (Table 1).

**Table 1:** Forearm ischemic exercise test Reference: [36]

	Lactate		Pyruvate	Ammonia
Normal response to ischemic exercise		↑ 3-5 times (normal)	↑ 3-5 times (normal)	↑ 5-10 times (normal)
Muscle glycogenosis		No increase or ↑ <2 times	No increase or ↑ <2 times	Normal
Mitochondrial defects		Normal or increased	Normal or increased	Normal
Lipid metabolism defects		Normal	Normal	Normal

Muscle biopsy plays a crucial role in diagnosing metabolic myopathies, as it allows histochemical and biochemical analysis [3,32-33]. Samples should be collected with a minimum interval of six months after the resolution of rhabdomyolysis, as muscle fibers, during their regeneration process, temporarily express a fetal isoenzyme immunologically distinct from mature myophosphorylase, which can result in a false-positive histochemical reaction for phosphorylase [31]. Specifically, in the case of McArdle's disease, muscle biopsy reveals sub-sarcolemmal glycogen deposits and, less commonly, intermyofibrillar deposits, shown by PAS-positive staining, while histochemical examination demonstrates the absence of myophosphorylase activity or, in some cases, residual activity below 10% [3,6].

Genetic testing as a CDT for McArdle's disease involves analyzing the most common mutations in the PYGM gene, which encodes myophosphorylase. Nevertheless, considering the genetic heterogeneity associated with McArdle's disease, the R50X mutation in the PYGM gene is the most prevalent mutation, especially in Caucasians, allowing genomic DNA analysis to detect the mutation in about 97 to 100% of cases, thus reducing the need for more invasive tests such as muscle biopsy [3-4,32-33].

McArdle's disease is typically diagnosed in the second or third decades of life due to the previously mentioned contingencies, including the causal relationship between physical activity and exercise intolerance symptoms manifesting only in adolescence [14,31,34].

However, there are cases where individuals remain asymptomatic for much of their lives, either because they avoid situations that trigger symptoms or because muscle damage remains subclinical. In these cases, symptomatic onset occurs when there is a need to utilize the anaerobic metabolic pathway [39].

Regarding treatment, since McArdle's disease presents a pattern of symptomatic manifestation in response to a stimulus, particularly physical exercise, treatment essentially focuses on symptomatic prevention, with a strong emphasis on ensuring available energy substrates [3].

In this regard, to increase resistance to muscle fatigue, several therapeutic approaches have been considered. These include adopting a diet rich in slowly absorbed carbohydrates (40%), with about 30% to 35% lipids and 25% to 30% proteins, combined with daily submaximal physical exercise [3,4,6]. This approach helps overcome the metabolic block imposed, thus reducing the symptoms associated with the disease [4,6].

Additionally, the intake of rapidly absorbed carbohydrates (sucrose) in the five minutes before exercise can improve exercise tolerance [3]. Recommended doses are 37 grams for adolescents and 18 to 20 grams for adults. Oral intake of fructose or glucose does not increase exercise tolerance and is associated with weight gain, which should be avoided in these patients [40,41].

Crisis prophylaxis, that is, preventive therapy, also involves avoiding vigorous exercise. However, the literature indicates that regular low-intensity physical activity has beneficial effects, as it optimizes circulatory capacity and energy metabolism using non-muscle energy substrates without increasing CK levels and potentially even reducing them [1,7-8,40,42].

Therapies with vitamin B6 supplements, creatine (to increase muscle membrane excitability), and angiotensin-converting enzyme (ACE) inhibitors appear to have some beneficial effects, although most of these therapeutic plans have inconsistent results [3,6-8,43].

Vitamin B6 supplementation is associated with improved muscle function, resulting in less muscle fatigue, as it is a cofactor of phosphorylase, which is reduced in these patients [3-4,8].

Studies have shown that creatine supplementation increases the capacity for isometric ischemic forearm exercise but does not improve isometric non-ischemic exercise with a low dosage (60 mg/kg/day). Conversely, daily high-dose creatine administration (150 mg/kg/day) has worsened exercise tolerance [1,8].

Disease severity has been correlated with the genotype of the ACE locus, where an insertion/deletion variant (D allele) is associated with increased ACE activity and is more commonly found in patients with more severe symptoms and lower exercise tolerance [3,44].

However, based on the observation that an increased number of copies of the D allele at the ACE locus is associated with greater disease severity, especially in women, treatment with ACE inhibitors appears to be associated with a better prognosis [45].

McArdle's disease does not usually represent an imminent life-threatening risk, although there are exceptional cases, such as neonatal presentation [3]. The prognosis is generally favorable, but effective self-management of the disease is essential to avoid major muscle injury, which can trigger rhabdomyolysis and acute renal injury, potentially leading to a less favorable prognosis [8].

## Conclusions

Although McArdle's disease is a rare condition, it should be considered in the differential diagnosis of rhabdomyolysis in all patients presenting with symptoms of exercise intolerance and/or recurrent myoglobinuria. This glycogen storage disease represents a pure myopathy caused by a genetic defect in the muscle isoform of myophosphorylase. The syndrome is characterized by exercise intolerance, manifested by myalgias, early muscle fatigue, contractions, and muscle weakness triggered by high-intensity or prolonged exercise. Less frequently, it can present with rhabdomyolysis, myoglobinuria, and acute renal injury.

To date, there is no effective gene therapy available, but patients may benefit from adopting a diet rich in slowly absorbed carbohydrates and an additional intake of rapidly absorbed carbohydrates before exercise. The practice of low-intensity aerobic exercise and supplementation with creatine, ACE inhibitors, and vitamin B6 seem to have beneficial effects, although their actual efficacy remains unproven.

## References

[1] Santalla A, Munguía-Izquierdo D, Brea-Alejo L (2014). Feasibility of resistance training in adult McArdle patients: clinical outcomes and muscle strength and mass benefits. Front Aging Neurosci.

[2] Schmid R, Mahler D (1959). Chronic progressive myopathy with myoglobinuria: demonstration of a glycolytic defect in muscle. J Clin Invest.

[3] Leite A, Oliveira N, Rocha M (2012). McArdle disease: a case report and review. Int Med Case Rep J.

[4] Oliveira G, Ferraz C, Coutinho P (2006). Doença de McArdle: caso clínico. Acta Pediatr Port.

[5] Costa R, Castro R, Costa A, Taipa R, Vizcaíno R, Morgado T (2013). Lesão renal aguda e rabdomiólise como apresentação da doença de McArdle. Acta Med Port.

[6] Sousa S, Gabriel JP, Pereira L (2009). De uma convulsão com rabdomiólise ao diagnóstico familiar de doença de McArdle. RN&C.

[7] Longo DL, Kasper DL, Jameson JL, Fauci AS, Hauser SL, Loscalzo J (2013). Medicina Interna De Harrison. 18º Edição, 2º Volume.

[8] Bollig G (2013). McArdle's disease (glycogen storage disease type V) and anesthesia - a case report and review of the literature. Paediatr Anaesth.

[9] Scalco RS, Chatfield S, Godfrey R (2014). From exercise intolerance to functional improvement: the second wind phenomenon in the identification of McArdle disease. Arq Neuropsiquiatr.

[10] Lucia A, Ruiz JR, Santalla A (2012). Genotypic and phenotypic features of McArdle disease: insights from the Spanish national registry. J Neurol Neurosurg Psychiatry.

[11] Kitaoka Y (2014). McArdle disease and exercise physiology. Biology (Basel).

[12] Andreu AL, Nogales-Gadea G, Cassandrini D, Arenas J, Bruno C (2007). McArdle disease: molecular genetic update. Acta Myol.

[13] Vieitez I, Teijeira S, Fernandez JM (2011). Molecular and clinical study of McArdle's disease in a cohort of 123 European patients. Identification of 20 novel mutations. Neuromuscul Disord.

[14] Bhavaraju-Sanka R, Howard J, Chahin N (2014). Atypical McArdle´s disease with asymmetric weakness and atrophy. SOJ Neurology.

[15] Park HJ, Shin HY, Cho YN, Kim SM, Choi YC (2014). The significance of clinical and laboratory features in the diagnosis of glycogen storage disease type v: a case report. J Korean Med Sci.

[16] Carlos CS, Oliveira VF, Saraiva LGF (2014). Glicogenoses: Uma Revisão Geral. Biosci J.

[17] Lorenzoni PJ, Lange MC, Kay CS, Scola RH, Werneck LC (2005). Motor nerve conduction study in McArdle disease: case report (Article in Portuguese). Arq Neuropsiquiatr.

[18] Felice KJ, Schneebaum AB, Jones HR Jr (1992). McArdle's disease with late-onset symptoms: case report and review of the literature. J Neurol Neurosurg Psychiatry.

[19] Kumar V, Abbas AK, Fausto N, Aster JC (2010). Patologia: Bases Patológicas Das Doenças. 8ª Edição.

[20] Porcelli S, Marzorati M, Belletti M, Bellistri G, Morandi L, Grassi B (2014). The "second wind" in McArdle's disease patients during a second bout of constant work rate submaximal exercise. J Appl Physiol (1985).

[21] Ørngreen MC, Jeppesen TD, Andersen ST (2009). Fat metabolism during exercise in patients with McArdle disease. Neurology.

[22] Galvão J., Gusmão L, Possante M (2003). Insuficiência renal e rabdomiólise induzidas por exercício físico. Rev Port Nefrol Hipert.

[23] Rossi LF, Ramos LAM, Ramos RR, Araújo ARC (2009). Rabdomiólise induzida por esforço físico intenso com altos níveis de creatinoquinase. Rev AMRIGS.

[24] Vanholder R, Sever MS, Erek E, Lameire N (2000). Rhabdomyolysis. J Am Soc Nephrol.

[25] Melli G, Chaudhry V, Cornblath DR (2005). Rhabdomyolysis: an evaluation of 475 hospitalized patients. Medicine (Baltimore).

[26] Giannoglou GD, Chatzizisis YS, Misirli G (2007). The syndrome of rhabdomyolysis: pathophysiology and diagnosis. Eur J Intern Med.

[27] Rosa NG, Silva G, Teixeira A, Rodrigues F, Araújo J (2005). Rabdomiólise. Acta Med Port.

[28] Zhang MH (2012). Rhabdomyolosis and its pathogenesis. World J Emerg Med.

[29] Khan FY (2009). Rhabdomyolosis: a review of the literature. Neth J Med.

[30] Pourmand R (2000). Metabolic myopathies: a diagnostic evaluation. Neurol Clin.

[31] Wortmann RL, DiMauro S (2002). Differentiating idiopathic inflammatory myopathies from metabolic myopathies. Rheum Dis Clin North Am.

[32] Darras BT, Friedman NR (2000). Metabolic myopathies: a clinical approach; part I. Pediatr Neurol.

[33] Berardo A, DiMauro S, Hirano M (2010). A diagnostic algorithm for metabolic myopathies. Curr Neurol Neurosci Rep.

[34] Arenas J, Martins MA (2003). Intolerancias metabólicas al ejercicio. Neurología.

[35] Kazemi-Esfarjani P, Skomorowska E, Jensen TD, Haller RG, Vissing J (2002). A nonischemic forearm exercise test for McArdle disease. Ann Neurol.

[36] Jensen TD, Kazemi-Esfarjani P, Skomorowska E, Vissing J (2002). A forearm exercise screening test for mitochondrial myopathy. Neurology.

[37] Hogrel JY, Laforêt P, Ben Yaou R, Chevrot M, Eymard B, Lombès A (2001). A non-ischemic forearm exercise test for the screening of patients with exercise intolerance. Neurology.

[38] Hanisch F, Eger K, Bork S, Lehnich H, Deschauer M, Zierz S (2006). Lactate production upon short-term non-ischemic forearm exercise in mitochondrial disorders and other myopathies. J Neurol.

[39] Dimaur S, Andreu AL, Bruno C, Hadjigeorgiou GM (2002). Myophosphorylase deficiency (glycogenosis type V; McArdle disease). Curr Mol Med.

[40] Lebo RV, Anderson LA, DiMauro S, Lynch E, Hwang P, Fletterick R (1990). Rare McArdle disease locus polymorphic site on 11q13 contains CpG sequence. Hum Genet.

[41] Vissing J, Haller RG (2003). The effect of oral sucrose on exercise tolerance in patients with McArdle's disease. N Engl J Med.

[42] Andreu A, Nogales-Gadea G, Cassandrini D, Arenas J, Bruno C (2007). McArdle disease: molecular genetic update. Acta Myol.

[43] Haller RG (2000). Treatment of McArdle disease. Arch Neurol.

[44] Martinuzzi A, Sartori E, Fanin M (2003). Phenotype modulators in myophosphorylase deficiency. Ann Neurol.

[45] Martinuzzi A, Liava A, Trevisi E (2008). Randomized, placebo-controlled, double-blind pilot trial of ramipril in McArdle's disease. Muscle Nerve.

